# Evaluation of a computer-assisted multi-professional intervention to address lifestyle-related risk factors for overweight and obesity in expecting mothers and their infants: protocol for an effectiveness-implementation hybrid study

**DOI:** 10.1186/s12889-020-8200-4

**Published:** 2020-04-15

**Authors:** Adrienne Alayli, Franziska Krebs, Laura Lorenz, Farah Nawabi, Anne-Madeleine Bau, Isabel Lück, Andrea Moreira, Judith Kuchenbecker, Elena Tschiltschke, Michael John, Stefan Klose, Benny Häusler, Christian Giertz, Ulrike Korsten-Reck, Stephanie Stock

**Affiliations:** 1 Institute of Health Economics and Clinical Epidemiology, University Hospital of Cologne (IGKE), Cologne, Germany; 2 Federal Centre for Health Education (BZgA), Cologne, Germany; 3 Platform Nutrition and Physical Activity (peb), Berlin, Germany; 4 Fraunhofer Institute for Open Communication Systems (FOKUS), Berlin, Germany; 5 Adiposity-Academy Freiburg, Freiburg, Germany

**Keywords:** Pregnancy, Overweight and obesity prevention, Lifestyle, Gestational weight gain, Multi-professional collaboration, Effectiveness, Implementation, Cost, Diet, Physical activity, Substance use.

## Abstract

**Background:**

The first 1000 days after conception are a critical period to encourage lifestyle changes to reduce the risk of childhood obesity and early programming of chronic diseases. A healthy lifestyle during pregnancy is also crucial to avoid high post-partum weight retention. Currently, lifestyle changes are not consistently discussed during routine health services in Germany. The objective of this study is to evaluate a novel computer-assisted lifestyle intervention embedded in prenatal visits and infant check-ups. The intervention seeks to reduce lifestyle-related risk factors for overweight and obesity among expecting mothers and their infants.

**Methods:**

The study is designed as a hybrid effectiveness-implementation trial to simultaneously collect data on the effectiveness and implementation of the lifestyle intervention. The trial will take place in eight regions of the German state Baden-Wuerttemberg. Region were matched using propensity score matching. Expecting mothers (*n* = 1860) will be recruited before 12 weeks of gestation through gynecological practices and followed for 18 months. During 11 routine prenatal visits and infant check-ups gynecologists, midwives and pediatricians provide lifestyle counseling using Motivational Interviewing techniques. The primary outcome measure is the proportion of expecting mothers with gestational weight gain within the recommended range. To understand the process of implementation (focus group) interviews will be conducted with providers and participants of the lifestyle intervention. Additionally, an analysis of administrative data and documents will be carried out. An economic analysis will provide insights into cost and consequences compared to routine health services.

**Discussion:**

Findings of this study will add to the evidence on lifestyle interventions to reduce risk for overweight and obesity commenced during pregnancy. Insights gained will contribute to the prevention of early programming of chronic disease. Study results regarding implementation fidelity, adoption, reach and cost-effectiveness of the lifestyle intervention will inform decisions about scale up and public funding.

**Trial registration:**

German Clinical Trials Register (DRKS00013173). Registered 3rd of January 2019, https://www.drks.de

## Introduction

Overweight and obesity are increasing worldwide [1]. More than one in two adults and nearly one in six children are overweight or obese in OECD countries [2]. In Germany 35.9% of the adult population are overweight and 18.1% are obese [3]. Among children and adolescents 15.4% are overweight and 5.9% are obese [4].

The high prevalence of overweight and obesity represents a key risk factor for non-communicable diseases, including cardiovascular diseases, diabetes, musculoskeletal disorders and some cancers [1]. As childhood overweight and obesity tend to persist into adulthood [5], early interventions are essential.

There is growing evidence that lifestyle factors in the first 1000 days after conception are important predictors of childhood overweight and obesity. Maternal gestational weight gain (GWG), smoking and diet have been identified as predictors during pregnancy [6–10]. Rapid infant weight gain, nicotine exposure and infant feeding practices have been identified as essential factors after birth [6, 10–13].

Human epidemiology and animal model studies suggest that exposure to these factors affects developmental processes, which program susceptibility to obesity and other chronic conditions manifesting later in life [14, 15]. Pregnancy and early infancy therefore represent a critical period for targeted prevention efforts.

Lifestyle changes initiated during pregnancy also produce benefits for expecting mothers. Evidence suggests that adequate GWG can avoid high post-partum weight retention and thus reduce the risk of overweight and obesity following pregnancy [16, 17].

Several preventive interventions addressing maternal lifestyle during pregnancy have been evaluated. Two meta-analyses show that diet and exercise interventions during pregnancy can effectively reduce excessive gestational weight gain [18, 19]. There is also evidence that professional-led educational interventions can increase uptake of breastfeeding [20]. A Cochrane review indicates that counseling interventions during pregnancy can effectively increase smoking cessation rates [21]. High post-pregnancy relapse rates call for strategies to promote continued abstinence post-partum, however [21, 22].

Lifestyle intervention trials initiated during pregnancy that continue during infancy are scarce [23–25]. They are heterogeneous, have methodological limitations and have produced mixed results [23, 24]. Few intervention studies provide evidence for beneficial effects on growth status of infants or children of obese women only [24].

Interventions targeting multiple lifestyle related risk factors hold promise for more effective childhood obesity prevention [10, 26]. So far, intervention studies targeting feeding, diet and physical activity behaviors in combination with prenatal nicotine exposure are lacking [23].

The GeMuKi project (acronym for “Gemeinsam Gesund: Vorsorge plus für Mutter und Kind” - Strengthening health promotion: enhanced check-up visits for mother and child) aims to incorporate a brief multifactorial lifestyle intervention into routine prenatal visits and infant check-ups. In Germany, these check-ups currently focus on early identification of diseases and developmental problems only. Existing guidelines for pre- and postnatal care mention that providers have a role in discussing modifiable lifestyle factors, but they do not specify content or format of lifestyle counseling [27].

Recent findings of the GeliS trial (acronym for "Gesund leben in der Schwangerschaft") conducted in the German state of Bavaria suggest that incorporating lifestyle counseling into routine prenatal health services is feasible and leads to high compliance rates [28]. The lifestyle intervention itself achieved only slight improvements in prenatal intake of food items, exclusive breastfeeding behavior and maternal post-partum weight development [29, 30]. By continuing lifestyle counseling after birth and utilizing theoretically underpinned Motivational Interviewing (MI) techniques, the GeMuKi intervention addresses some limitations of the GeliS intervention. In addition, the GeMuKi intervention includes a novel shared telehealth platform to support multi-professional providers in the counseling process with a corresponding App for intervention participants.

The objective of this study is to examine effectiveness of the GeMuKi intervention and explore its potential for widespread implementation. It will answer the following research questions:

- Does the GeMuKi intervention effectively improve lifestyle-related risk factors for overweight and obesity in expecting mothers and their infants compared to routine practice?
- How does implementation of the GeMuKi intervention take place in practice? What factors facilitate or hinder successful implementation during routine prenatal visits and infant check-ups?
- What costs, health service use and consequences are associated with the GeMuKi intervention from a public payer perspective? How do these compare to routine health practice?

## Methods/design

### Study design

A hybrid effectiveness-implementation trial (Type II) is being used to simultaneously collect data on the effectiveness and implementation of the GeMuKi lifestyle intervention [31, 32]. This design was selected because there is strong evidence that interventions during pregnancy can effectively improve lifestyle-related risk factors, research indicates that lifestyle counseling during routine check-up visits is feasible in Germany and evidence on implementation of lifestyle interventions during pregnancy is scarce. The GeMuKi intervention comprises various components previously identified to enhance lifestyle counseling during pregnancy. To our knowledge, effectiveness of these components has not been evaluated in combination, yet.

**Fig. 1:**
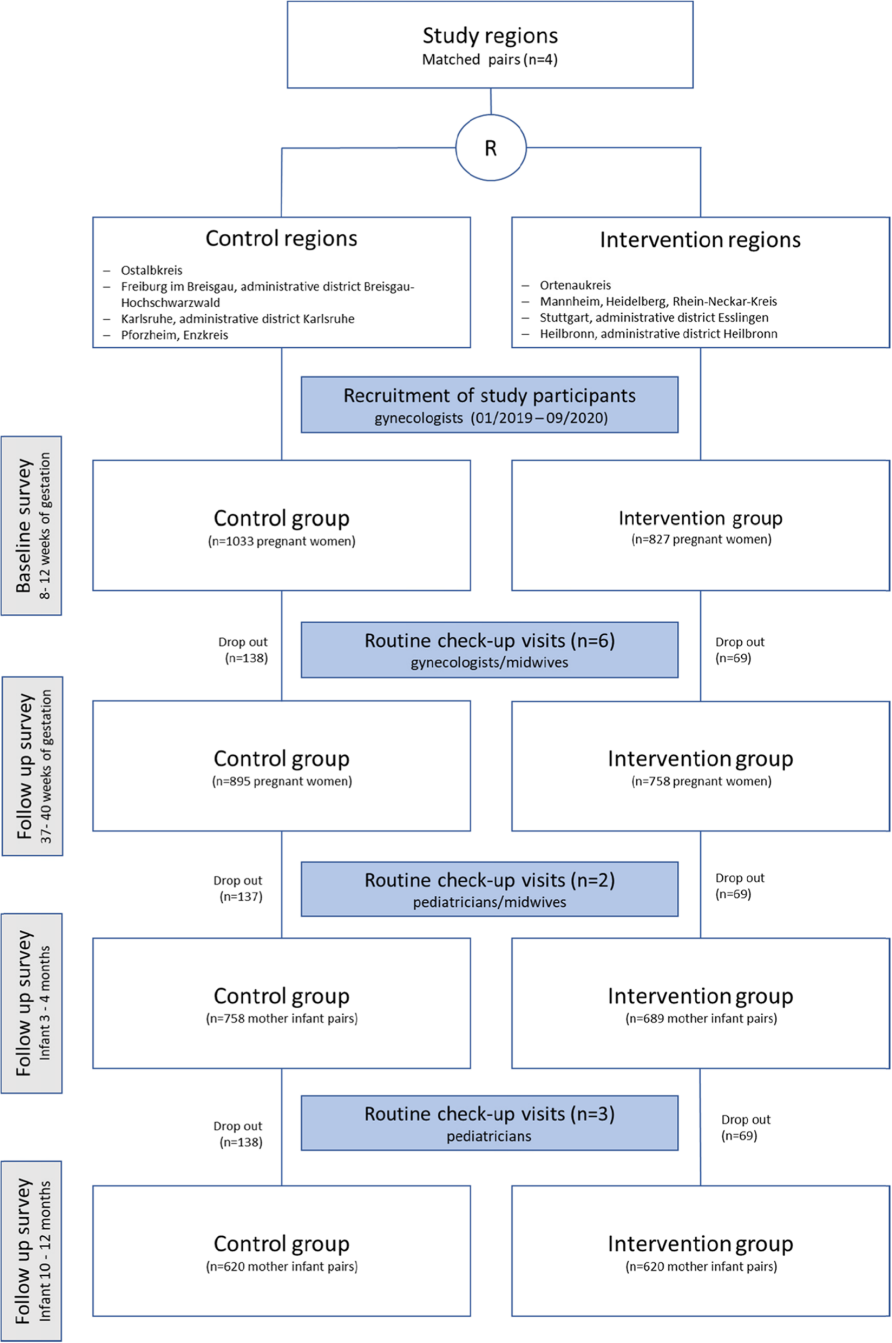
Study design flow chart

The trial has two arms (see Fig. 1). In the intervention arm gynecologists, midwives and pediatricians carry out the GeMuKi lifestyle intervention during routine prenatal visits and infant check-ups. In the control arm they provide care as usual. The study takes place in both urban and rural areas within the German state Baden-Wuerttemberg. To reduce discrepancies between study regions intervention and control regions were matched into pairs using propensity score matching. Matching was conducted immediately after the project kick off in October 2017 to provide enough time for enrollment of multi-professional providers and for conducting implementation training in the intervention regions before commencing recruitment of study participants. Matching was based on average income per capita, the number of births among persons insured by BARMER (i.e. the statutory health insurer agreeing first to participate in the GeMuKi project) and the number of gynecologists in the study regions. This resulted in four matched study region pairs, which were randomized into intervention and control regions.

Data regarding effectiveness and implementation will be collected at multiple time points over an 18-month study period (see Table 2).

### Recruitment procedure

Recruitment of multi-professional providers commenced in April 2018 and continues until December 2019. For this purpose, informational meetings are being conducted in the study regions. Regional opinion leaders are attending these meetings to raise awareness of the GeMuKi project and promote participation from a user-perspective. Additionally, the project is advertised through professional organizations, journals, conference presentations and through contacting providers directly over the phone and during personal visits.

Recruitment of study participants commenced in January 2019 and continues until September 2020. It takes place during routine prenatal visits conducted in participating gynecologist practices before 12 weeks of gestation. Gynecologists determine eligibility of pregnant women using pre-defined in- and exclusion criteria. They provide eligible women with a project brochure and additional information about the study. For each study participant, who enrolls in the study, gynecologists receive an expense allowance of 20€.

### In- and exclusion criteria

Pregnant women are eligible to participate, if they provide informed consent, are ≥18 years old, are < 12 weeks of gestation at recruitment, are proficient in German language and are enrolled in one of the participating gynecologist practices. To participate in the study, pregnant women also require a health insurance plan from BARMER or from one of the following statutory health insurers, who became project partners upon commencement of the GeMuKi project: AOK Baden-Württemberg, Techniker Krankenkasse and through GWQ Service Plus: Audi BKK, BAHN-BKK, Bertelsmann BKK, BIG direkt gesund, BKK Deutsche Bank AG, BKK Schwarzwald-Baar-Heuberg, BKK Voralb HELLER *Index* LEUZE, Daimler BKK, Die Schwenninger Krankenkasse, energie-BKK, Heimat Krankenkasse, Salus BKK, SBK Siemens-Betriebskrankenkasse, SECURVITA Krankenkasse.

Pregnant women who screen positive for depression (i.e. defined as a sum score of > 9 or a score = 3 on item 10 of the Edinburgh Postnatal Depression Scale) are excluded from the study. They are referred to information about the ‘Mind: Pregnancy’ trial, which takes place simultaneously in the same study regions [33]. It evaluates an intervention to reduce psychological stress during pregnancy. This procedure aims to reduce risk of bias that could be introduced by co-interventions.

### Multi-professional computer-assisted lifestyle intervention

**Fig. 2:**
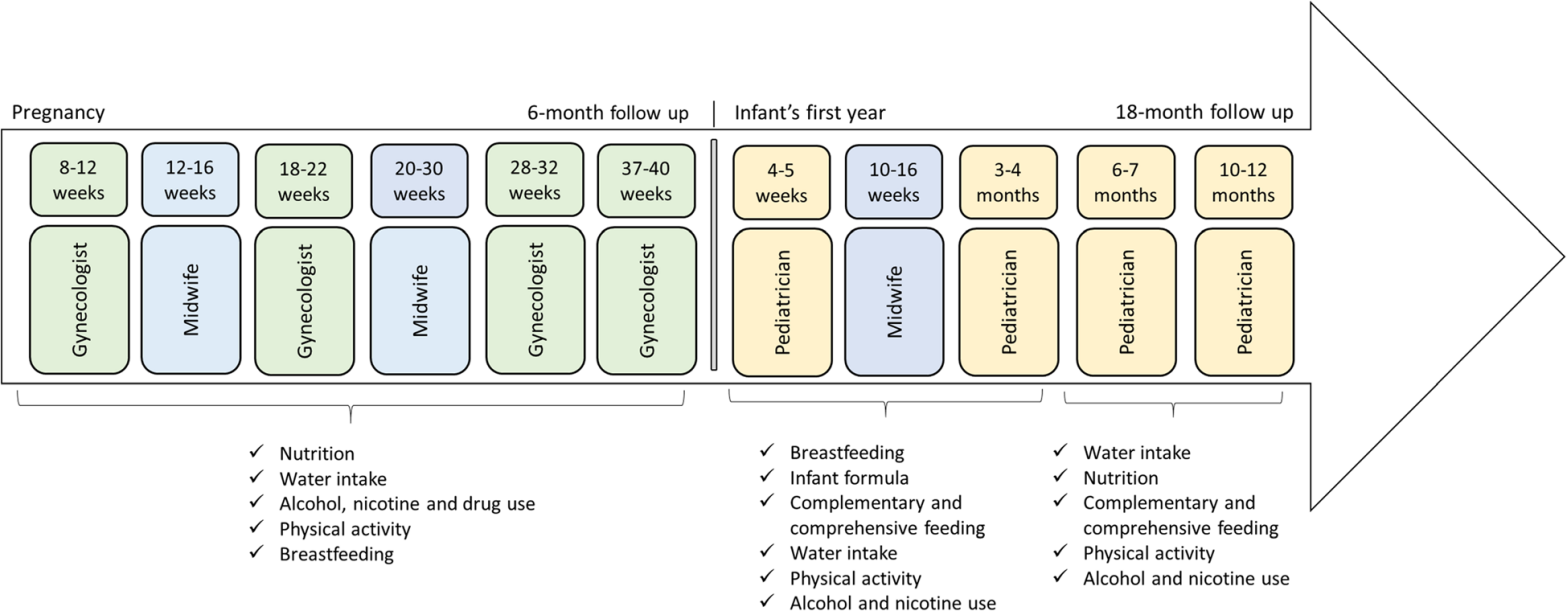
Topics addressed by the GeMuKi intervention during routine prenatal visits and infant check-ups

The development of the GeMuKi intervention has been informed by experiences from the project 9 + 12 [34] and the GeliS study [28–30]. It aims to positively influence lifestyle-related risk factors of expecting mothers and their infants. The GeMuKi intervention is designed as a series of brief (approximately 10 min) counseling sessions performed by gynecologists, midwives and pediatricians during 11 prenatal and infant check-ups (see Fig. 2). The counseling sessions cover topics relevant during pregnancy and the infant’s first year relating to diet, physical activity, breastfeeding, and substance use. Figure 2 provides an overview of the topics addressed over the course of the GeMuKi lifestyle intervention. The topics are based on recently updated national recommendations developed by a multidisciplinary scientific task force [35, 36].

Traditionally, behavioral interventions aiming at lifestyle changes rely on providing information and advice. This has proven to be less successful compared to approaches using elements of Motivational Interviewing (MI) to improve communication by health professionals [37, 38].

The GeMuKi intervention takes into consideration that communication of providers should be sensitive to expecting mothers’ health literacy in order to have a positive impact on behavior change. Therefore, multi-professional providers carrying out the GeMuKi intervention receive communication skills training. In addition to the content of the lifestyle intervention itself, the training covers MI techniques. MI is a client-centered counseling approach designed to enhance motivation for behavioral change by helping clients explore and resolve ambivalence [39].

A key element of MI used in the GeMuKi intervention is agenda mapping. Multi-professional providers employ agenda mapping to focus on a specific topic for lifestyle change (see Fig. 2). For this purpose, they use key message cards with pictograms developed by the Platform Nutrition and Physical Activity (peb) and experienced MI trainers.

After a participant has chosen a topic for lifestyle change, the provider continues the conversation using open-ended questions and then reacts to the participant’s answers using reflective listening techniques. Guided by the provider, participants set SMART (Specific Measurable Achievable Reasonable Time Bound) goals for lifestyle change, which can be accomplished until the next check-up visit.

Another objective of the GeMuKi intervention is to increase the level of cooperation between gynecologists, pediatricians and midwives. To achieve this, a novel telehealth platform was developed, which assists providers in the counseling process and enables them to communicate with each other.

#### Telehealth platform GeMuKi-Assist

**Fig. 3:**
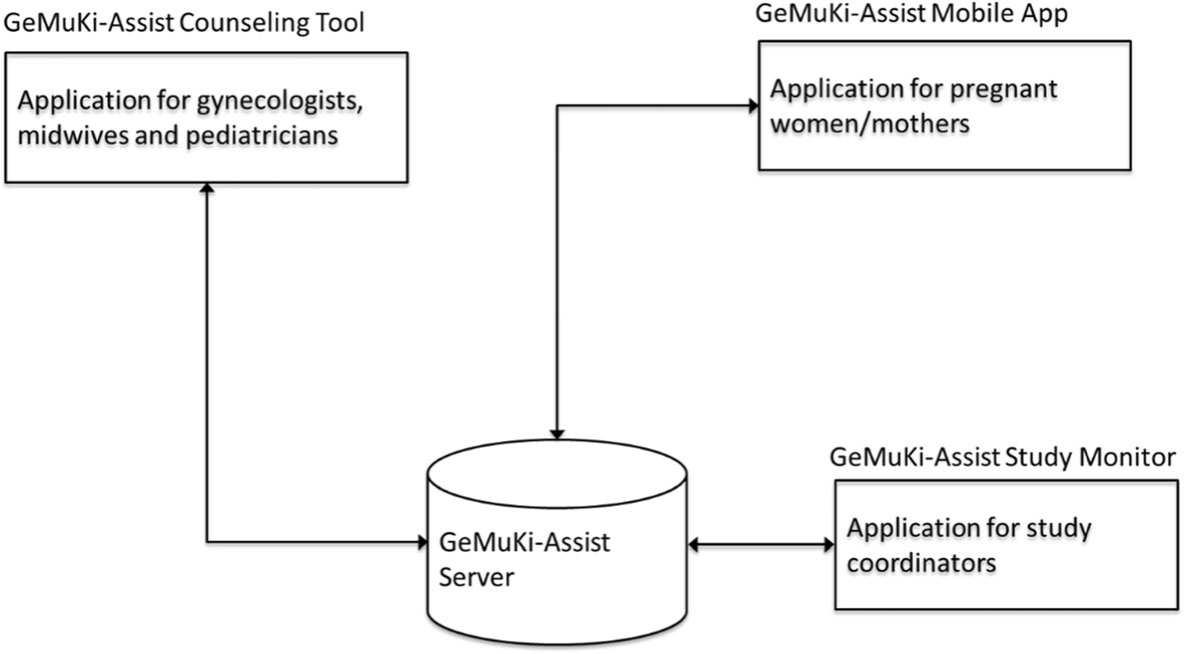
Overview GeMuKi-Assist Telehealth platform

The telehealth platform GeMuKi-Assist has the objective to facilitate cooperation between providers and enhance continuity of lifestyle counseling. It consists of the GeMuKi-Assist Counseling Tool, GeMuKi-Assist App, GeMuKi-Assist Study Monitor and the GeMuKi-Assist Server (see Fig. 3).

Providers and trained practice staff in both intervention and control regions use the GeMuKi-Assist Counseling Tool to enter data routinely documented in the maternity and child medical record booklets. In the intervention regions these data are used to create a GWG curve showing the development of GWG for each individual study participant in relation to the recommended range. The infants’ weight progression is displayed by means of percentile curves (see Additional file 1). Providers in the intervention regions also have access to key messages and guiding questions (i.e. standardized content) to support them in carrying out the GeMuKi intervention according to protocol and in alignment with MI techniques. They can also document goals for lifestyle change participants want to accomplish until the next check-up visit and have an option to enter notes regarding individual participants. To ensure continuity of the counseling, this information can be accessed by multi-professional providers involved in the counseling process. Individual goals for lifestyle change entered into the counseling tool are automatically send to the GeMuKi-Assist App as a reminder for study participants.

The GeMuKi-Assist App aims to support intervention group participants in performing lifestyle changes. It provides an overview of individual goals formulated during lifestyle counseling and sends automatic reminders for encouragement (push notifications). The App includes links to reliable sources of information (e.g. institutions providing health education) as well as services and supports available in the region (e.g. psychotherapists and dieticians). An option to conduct automated google keyword searches (e.g. lactation consultant and smoking cessation classes) is also included (see Additional file 1). In addition to these features, which are only available for participants in the intervention group, all participants can use the App for creating personal notes and completion of the electronic surveys in the study.

The GeMuKi-Assist Study Monitor supports the research process alongside the GeMuKi intervention. It is used to create user profiles for providers and study participants and for assigning study participants to corresponding multi-professional providers. Study coordinators also use the tool to monitor the data collection process. Automatic alerts from the GeMuKi-Assist server inform them for instance about incomplete data from study participant surveys and data entries in the counseling tool (see Additional file 1).

The GeMuKi-Assist Server handles and saves the data derived from the mobile App, the counseling tool and the study monitor in one central database. Access is controlled for different user groups, who must authorize themselves before accessing the data.

### Implementation strategy

To encourage uptake of the GeMuKi-intervention and implementation as planned an implementation strategy is being used consisting of the three elements: (1) a one-day training for gynecologists, midwives, pediatricians and practice assistants; (2) support by regional study coordinators in participating practices and (3) funding of novel tasks associated with the lifestyle intervention.

The one-day training is conducted before initiating the GeMuKi lifestyle intervention. It covers the basics of MI and the previously mentioned updated national recommendations for a health-promoting lifestyle during pregnancy and the infant’s first year. The training material includes a PowerPoint presentation, key message cards as well as brochures and stickers for the maternity and child medical record booklets. The presentation provides information on the purpose of the lifestyle intervention and key messages for a health-promoting lifestyle. It also summarizes the most relevant aspects of the evaluation study conducted alongside the intervention. In addition, the fundamentals of MI are introduced and the implementation of the GeMuKi intervention using selected MI elements explained. Knowledge of theoretical concepts are applied practically through role-play exercises and reinforced by videos with MI examples. The training also covers how to use the GeMuKi-Assist Counseling Tool. The training is carried out by experienced MI trainers from the Healthy Start-Young Family Network (Gesund ins Leben-Netzwerk Junge Familie). The training materials were developed based on the content of the curriculum of the Healthy Start-Young Family Network [40] and additional literature [41–43].

Regional study coordinators provide ongoing support to participating providers over the phone and during regular practice visits. They conduct a hands-on introduction to GeMuKi-Assist in the participating providers’ practices in both intervention and control regions and answer questions to help solve technical issues with GeMuKi-Assist and other local implementation challenges. They also provide information and advice to encourage protocol compliance (e.g. regarding weighing during pregnancy and flawless documentation). Furthermore, they perform data management. In case of missing data or data error, they contact the respective providers.

All providers participating in the study receive funding for implementing the GeMuKi intervention. They sign a contract with the participating health insurers and the Association of Statutory Health Insurance Physicians of Baden-Württemberg (KVBW). This contract forms the legal basis for the billing process. Providers in the intervention regions can bill 15 € per lifestyle counseling session. Providers in both the intervention and control regions can bill 5 € per documentation in GeMuKi Assist. Gynecologists and pediatricians in the intervention regions can receive up to 80 € and midwives up to 60 € per study participant when they carry out all counseling sessions in the study period (see Fig. 2).

### Data sources

Data will be collected at various points in time using multiple methods. Data sources include an electronic survey completed by study participants in the GeMuKi-Assist App at four points in time, data entered into the GeMuKi-Assist counseling tool during routine prenatal visits and infant check-ups, (focus group) interviews with multi-professional providers and intervention participants, statutory health insurance claims data and documents. At baseline, study participants also complete a short paper survey including demographic questions.

The selection of data sources was guided by the RE-AIM framework, which has been developed for evaluation of effectiveness and implementation of interventions in real-world settings [44, 45]. Table 1 provides a summary of constructs that will be measured for each dimension of the RE-AIM framework and data sources used.

**Table 1:** Data sources and measured constructs aligned with RE-AIM dimensions

Dimension	Definition	Measured construct	Data source
Reach	The absolute number, proportion, and representativeness of individuals who are willing to participate in an initiative, intervention, or program.	Number and characteristics of participants and non-participants, reasons for non-participation	Administrative data in GeMuKi-Assist, focus groups with multi-professional providers, paper survey
Effectiveness	The impact of an intervention on important outcomes, including potential negative effects, quality of life, and economic outcomes.	Proportion of participants with excessive weight gain, infant body composition and weight development	Administrative data in GeMuKi-Assist
		Maternal lifestyle, knowledge, infant feeding, infant diet and physical activity	Electronic survey
Adoption	The absolute number, proportion, and representativeness of settings and intervention agents (people who deliver the program) who are willing to initiate a program.	Proportion and characteristics of participating multi-professional practices, reasons for non-participation and drop-out of practices	Administrative data in GeMuKi-Assist, documents and publicly available statistics
Implementation	*Setting level:* the intervention agents’ fidelity to the various elements of an intervention’s protocol, including consistency of delivery as intended and the time and cost of the intervention.	Implementation of brief lifestyle advice intervention (how and by whom?)	Focus groups with multi-professional providers,
		Intervention costs: human resources and time, health service use, implementation costs and training	Administrative data in GeMuKi-Assist, interviews with study participants, social health insurance claims data, documents
		Utilization of the GeMuKi Assist Counseling Tool, local adaptations of the intervention	Focus groups with multi-professional providers, interviews with study participants, administrative data in GeMuKi-Assist
	*Individual level*: the clients’ use of the intervention strategies.	Utilization of GeMuKi-Assist App, goal setting, links etc. Attainment of lifestyle change goals	Interviews with study participants, administrative data in GeMuKi-Assist
Maintenance	*Setting level:* the extent to which a program or policy becomes institutionalized or part of the routine organizational practices and policies.	Providers becoming experienced in delivering lifestyle advice, lifestyle advice becoming a routine component of practice processes	Focus groups with multi-professional providers, administrative data in GeMuKi-Assist
	*Individual level:* the long-term effects of a program on outcomes after 6+ months after the most recent intervention contact.	Maintenance of lifestyle changes and weight, drop out of study participants	Administrative data in GeMuKi-Assist, electronic survey

### Measures to assess effectiveness of the lifestyle intervention

Outcomes used to assess are described below. Table 2 provides a summary of the points of measurement and data collection methods.

**Table 2:** Outcome measures at baseline and follow up

	Pregnancy				Infant’s first year					
	8–12 weeks	18–22 weeks	28–32 weeks	37–40 weeks	At birth	3–10 days	4–5 weeks	3–4 months	6–7 months	10–12 months
Maternal weight ^a^	x	x	x	x						x
Maternal physical activity	x			x				x		
Maternal smoking	x			x				x		x
Maternal alcohol use	x			x				x		x
Maternal diet	x			x				x		
Maternal knowledge	x			x				x		x
Breastfeeding ^a^							x	x	x	x
Infant weight and length ^a^					x	x	x	x	x	x
Infant nutrition										x
Infant physical activity								x		x

Notes: a = data are routinely collected and transferred into GeMuKi-Assist during check-up visits, all other measures are collected by an electronic self-report survey. Please note that this table only includes check-up visits, in which providers assess the specified outcomes

#### Maternal weight

During every prenatal visit maternal weight is routinely measured and documented in the maternity record booklet (see Table 2). GWG is calculated as the difference between self-reported pre-pregnancy weight documented during the first prenatal visit and weight at the last prenatal visit.

Excessive GWG is defined according to recommendations of the Health and Medicine Division of the National Academies of Science, Engineering and Medicine (previously known as Institute of Medicine, IOM). These recommendations differ depending on pre-pregnancy Body Mass Index (BMI). For underweight women (BMI < 18.5) the recommended weight gain ranges from 12.5 to 18 kg, for normal weight women (BMI = 18.5–24.9) from 11.5 and 16 kg, for overweight women (BMI = 25–29.9) from 7 to 11.5 kg and for obese women (BMI ≥ 30) from 5 to 9 kg [46]. Weight gain above the recommended range is classified as excessive GWG. This definition of excessive GWG is similar to the definition used in German guidelines, which currently recommend a maximum weight gain of 16 kg for normal weight women and a maximum of 10 kg for overweight and obese women [36]. To assess postnatal weight-retention, maternal weight data will also be collected 1 year after birth.

#### Maternal lifestyle behaviors

Physical activity behavior during pregnancy will be measured using the Pregnancy Physical Activity Questionnaire (PPAQ) [47]. Maternal smoking behavior and alcohol consumption will be measured using questions from the German Health Interview and Examination Survey for Children and Adolescents (KIGGS) [48]. Dietary behavior will be assessed with a modified version of the Food Frequency Questionnaire used in the German Health Examination Survey for Adults (DEGS), which measures frequency and portion size of the main food groups consumed over the past 4 weeks [49].

#### Maternal knowledge

To assess the ability of the lifestyle intervention to increase maternal knowledge of health promoting lifestyle aspects addressed during brief counseling, the research team developed specific knowledge questions. These questions are based on key messages included in the previously mentioned national recommendations for a health-promoting lifestyle during pregnancy and the infant’s first year [35, 36]. Data on study participants’ health literacy will be collected as part of a separate study component, which will be reported elsewhere.

#### Infant weight development and body composition

Infant weight and length will be routinely assessed during infant check-ups. Infant BMI will be calculated and compared with age-specific reference values. The German Kromeyer-Hauschild reference system [50] will be used, because national reference data are more suitable for diagnosis of childhood overweight and obesity [4, 51]. To allow for comparisons with international research, the research team will also compare infant weight and length measures with WHO Growth Standards [52].

#### Infant feeding, diet and physical activity

Breastfeeding will be routinely documented in the GeMuKi-Assist counseling tool during infant check-ups. At the age of 10 to 12 months study participants will complete a modified version of the food frequency questionnaire used in the German Health Interview and Examination Survey for Children and Adolescents (KIGGS) [53]. It measures frequency and portion sizes of main food groups infants consumed over the past 4 weeks. Additionally, parental feeding practices will be examined with single items from the Comprehensive Feeding Practices Questionnaire (CFPQ) [54]. Study participants will also complete several questions on their infants’ physical activity behavior developed by the research team.

### Evaluation of the implementation process

To gain insights into the implementation process, the study team will examine which components of the lifestyle intervention are implemented as planned and which components are being modified. For this purpose, focus groups and interviews with multi-professional providers and study participants will be carried out. Additionally, data entered into the GeMuKi-Assist Counseling Tool will be analyzed. Among other variables, the research team will analyze counseling contents, characteristics of participating providers, characteristics of expecting women and infants reached by the intervention and the total number of lifestyle counseling sessions provided. Finally, documents will be analyzed, such as minutes taken during implementation training.

Qualitative interviews and focus groups will provide insights into factors facilitating and hindering implementation from the perspective of providers and participants in the lifestyle counseling. These qualitative data will also shed light on contextual factors influencing the implementation process and outcomes for expecting mothers or their infants. To examine dynamic changes over time the research team will conduct interviews and focus groups both at the beginning and the end of the implementation process.

The evaluation of the implementation process will be informed by the Tailored Implementation for Chronic Diseases (TICD) checklist. This checklist is based on a synthesis of frameworks and taxonomies of determinants of professional practice [55]. It identifies determinants that influence professional practice in seven domains: guideline factors, individual health professional factors, patient factors, professional interactions, incentives and resources, capacity for organizational change, social political and legal factors. The checklist will guide the choice of measures used to understand factors influencing adoption, implementation and maintenance of the GeMuKi intervention by multi-professional providers.

### Economic evaluation

A cost-consequence analysis will be performed, because the GeMuKi intervention seeks to modify multiple outcomes in expecting mothers, their infants and at the system level. Cost-consequence analyses compare costs and consequences of alternatives in a disaggregated manner [56]. This provides greater transparency to decision makers, who want to weigh multiple aspects against each other [57, 58].

The analysis will be conducted from a health insurance perspective. Cost components considered in the analysis include intervention costs, health service use and implementation costs. Intervention and implementation costs will be calculated based on documentation of personnel time and other resources used. Service use will be calculated using social health insurance claims data. These data include in- and outpatient treatment, medication use, aids and remedies, use of preventive services and sick leave periods. Outcomes considered in the analysis will include the above described lifestyle-related risk factors for overweight and obesity in expecting mothers and their infants. Additionally, outcomes at the system-level will be considered, such as changes in collaboration practices between multi-professional providers. These will be derived from qualitative data analyses conducted to gain understanding of implementation processes.

### Sample size calculation

GWG was used as primary outcome for the sample size calculation, because healthy GWG is discussed with all expectant mothers participating in the lifestyle intervention. The brief lifestyle intervention is assumed to reduce the proportion of study participants with excessive gestational weight gain by 10%. Similar interventions have achieved a reduction in the proportion of excessive weight gain of around 20% [18, 59]. The target was set lower in this study, because the lifestyle intervention is implemented in a routine health service setting with less stringent inclusion criteria. To detect a 10% reduction in excessive gestational weight gain with a power of 80%, an alpha of 0.05 and an ICC of 0.05 a sample of *n* = 1240 pregnant women is required. This number was increased to *n* = 1860 to account for a drop-out rate of 25% in the intervention group and a 40% drop-out rate in the control group (see Fig. 1).

### Data analyses

The data entry fields in the GeMuKi-Assist Counseling Tool and electronic surveys collected through the GeMuKi-Assist App are predefined to allow for plausible data only. Additional plausibility checks will be performed before commencing data analysis. Analyses of these quantitative data using descriptive statistics, statistical tests and regression models will be conducted in SPSS and R. Analyses for all primary and secondary outcomes will follow an intention-to-treat principle, which compares the intervention arm to the control arm, without regard to intervention completion or compliance. Mixed effects models will be used to account for the clustered structure of the data. Multiple imputation methods will be used to deal with missing values. Exploratory analyses will be performed to explore intervention outcomes for subgroups of study participants, e.g. according to SES and migration background.

All focus groups and interviews will be audio-recorded and transcribed verbatim. Qualitative analysis of focus groups, interviews and documents will be carried out in MAXQDA using a framework analysis approach [60]. Two multidisciplinary researchers will conduct coding independently and discuss discrepancies. The principle of triangulation will be applied continuously to test validity through comparing information from different data sources.

To provide a better understanding of the overall process of implementation and gain insights into possible interactions between implementation and effectiveness of the GeMuKi intervention the research team will conduct integrated data analyses combining qualitative and quantitative data sources.

## Discussion

This study will evaluate a brief counseling intervention to reduce lifestyle-related risk factors of overweight and obesity among expectant mothers and their infants. The GeMuKi intervention is innovative, as it combines several components that have been identified to enhance lifestyle counseling during pregnancy.

First, the lifestyle counseling is integrated into routine prenatal visits and infant check-ups. This puts a smaller burden on participants than add-on approaches [61] and provides a low threshold approach to reach expecting mothers and their infants. According to most recent estimates almost 90% of expecting mothers in Germany regularly attend prenatal visits [62] and over 95% of infants attend infant check-ups during the first year of life [63].

Second, lifestyle counseling is tailored to individual intervention participants. A tailored approach that recognizes individual differences in motivation, knowledge, needs and circumstances is recommended, because one-size fits all approaches have shown to be less effective in preventing overweight and obesity [61, 64]. The GeMuKi intervention consists of a series of brief counseling sessions using MI techniques. MI is a person-centered counseling approach, which encourages active involvement of intervention participants in the behavior change process. As evidenced by systematic reviews, MI has effectively promoted different health behaviors [65, 66] and has been associated with lifestyle changes in the long-term [67].

Third, providers implementing the GeMuKi intervention, will receive training in applying MI techniques. This will address needs expressed by professionals providing pre- and postnatal care to improve communication skills to discuss the sensitive topic of obesity and gestational weight gain [68–71].

Fourth, lifestyle counseling in the GeMuKi intervention will be supported by the novel telehealth platform GeMuKi-Assist. It includes a counseling tool for documentation and collaboration between multi-professional providers, an App for study participants with supporting information to encourage attainment of lifestyle change goals and a study monitor to support the evaluation study. An increasing body of evidence suggests that when used as an adjunct to face-to-face counseling methods computer and communication technology can be an effective tool to achieve lifestyle behavior changes, also among women with lower socio-economic status [72, 73].

Finally, the GeMuKi lifestyle counseling will be provided continuously over an 18-month period. This is in line with previous research findings, demonstrating that longer duration of lifestyle interventions result in more effects [74, 75].

The GeMuKi intervention will be evaluated in eight regions of the German state Baden-Wuerttemberg. To support implementation as planned, a comprehensive implementation strategy has been developed. It includes a training curriculum and funding scheme, which can be scaled up, in case the intervention proves to be effective. This evaluation study is designed to provide insights for policy makers at the German Federal Joint Committee (G-BA), who will decide about roll-out and public funding of the intervention on a federal scale.

### Strengths of the study

The effectiveness-implementation hybrid design will concurrently provide insights into effectiveness of the GeMuKi intervention and the process of implementation. It combines design features from a pragmatic clinical trial with concepts from implementation research in order to facilitate a more rapid translation of research evidence into practice [31]. Guided by the RE-AIM framework, various data sources will be used to add further context to findings on effectiveness of the GeMuKi intervention. The study will provide information about factors that influence adoption of the intervention by multi-professional providers, reach of the target group, implementation fidelity and costs. Both from the perspective of providers as well as intervention participants the study will identify ways to optimize the intervention to enhance effectiveness, client satisfaction and ease of implementation.

The 18-month follow up is a second strength of this study. Expecting mothers will be included in the study before 12 weeks of gestation and will participate in the GeMuKi intervention until 1 year after birth. The study findings will add to the limited evidence from intervention studies aimed at reducing risk of childhood overweight and obesity, which are commenced during pregnancy and continued after birth [23–25]. They will increase our understanding of effective early intervention strategies to prevent early programming of chronic disease.

### Challenges and limitations

Execution of this study protocol involves several challenges. Embedding the GeMuKi intervention into routine care may pose a challenge for providers, who already have limited time during busy patient schedules [61]. To support providers in conducting the lifestyle counseling efficiently and as planned, the GeMuKi-Assist Counseling Tool includes various supports for providers, such as example questions to discuss with women.

Detecting expected effects of the GeMuKi intervention will require a large sample size. To address this challenge, multiple recruitment strategies will be used. To encourage intervention uptake by multi-professional providers the research team will involve regional opinion leaders among professional groups. Additionally, a relatively high drop-out rate was assumed in the power calculation.

An intervention provided in health service settings can only have a limited impact on individual lifestyle behaviors. Important other determinants in the social, physical and economic environment are not directly addressed by the GeMuKi intervention. Study participants can only be referred to additional supports and resources available in the community. Hence, the GeMuKi intervention can only be one element in an integrated, system wide approach required for successful obesity prevention.

## Supplementary information

**Additional file 1.** GeMuKi-Assist Telehealth Platform – additional information and illustrations.

## Data Availability

Not applicable.

## Abbreviations

BMI: Body Mass Index
GeMuKi: Gemeinsam Gesund Vorsorge Plus für Mutter und Kind (Strengthening health promotion: enhanced check-up visits for mother and child)
GWG: Gestational weight gain
MI: Motivational Interviewing
OECD: Organisation for Economic Co-operation and Development
