# Supplementary material for: Evaluation of a computer-assisted multi-professional intervention to address lifestyle-related risk factors for overweight and obesity in expecting mothers and their infants: protocol for an effectiveness-implementation hybrid study
Source: BMC Public Health. 2020 Apr 15;20:482. doi: 10.1186/s12889-020-8200-4 (PMC7158079; doi:10.1186/s12889-020-8200-4)
Supplement: Supplementary file 1 — Additional file 1. GeMuKi-Assist Telehealth Platform – additional information and illustrations. [file 12889_2020_8200_MOESM1_ESM.docx]

**Additional file 1.** GeMuKi-Assist Telehealth platform – additional information and illustrations

The GeMuKi Assist Telehealth Platform complies with the EU General Data Protection Regulation (GDPR). Internet communication channels are encrypted and personal data is stored encrypted in the database on the server. The GeMuKi-Assist Server is hosted in a certified data center based on the ISO/IEC 27001 standard. The figures below illustrate several key features of the telehealth platform.


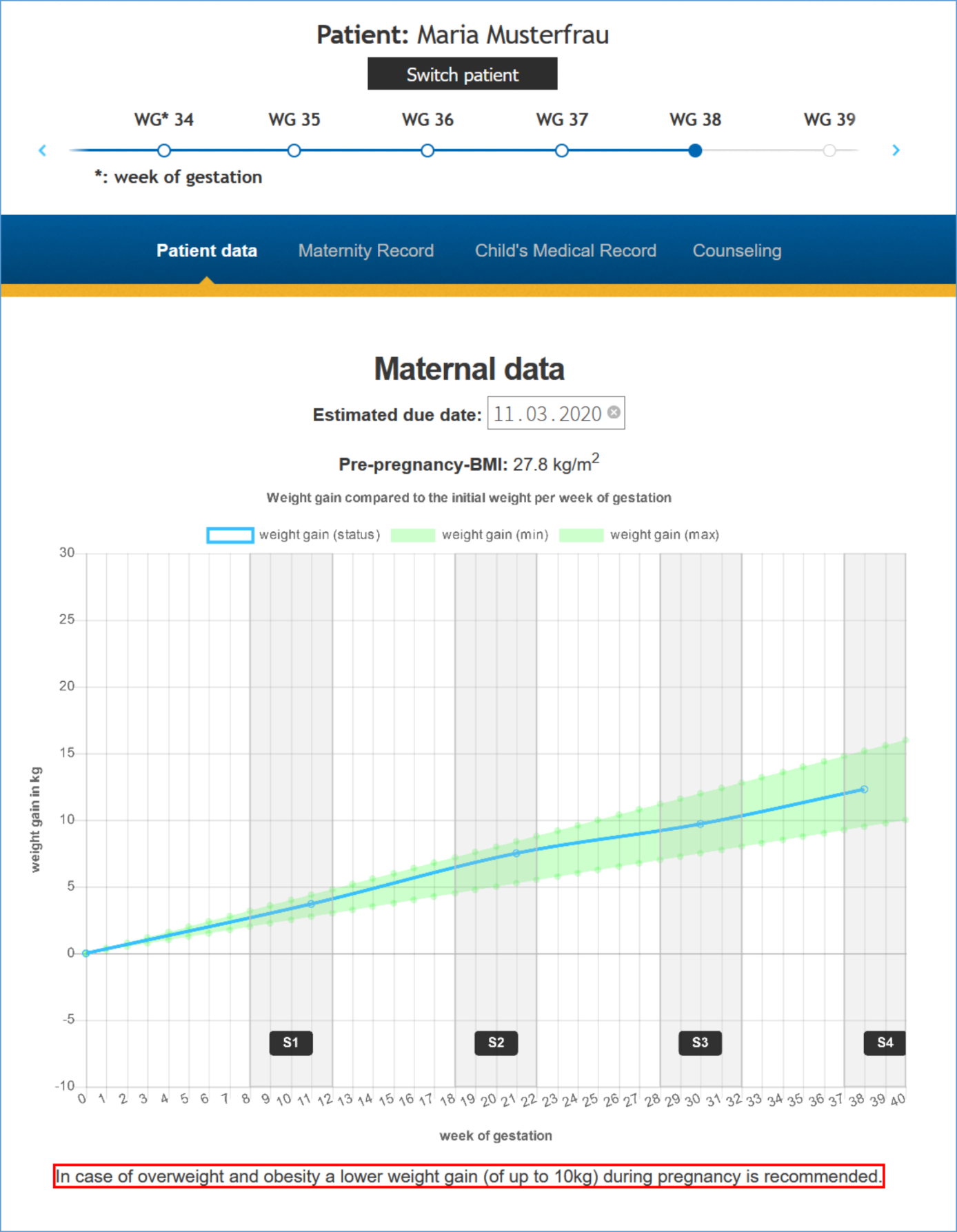


**Figure 1** GWG curve displayed in the GeMuKi-Assist counseling tool


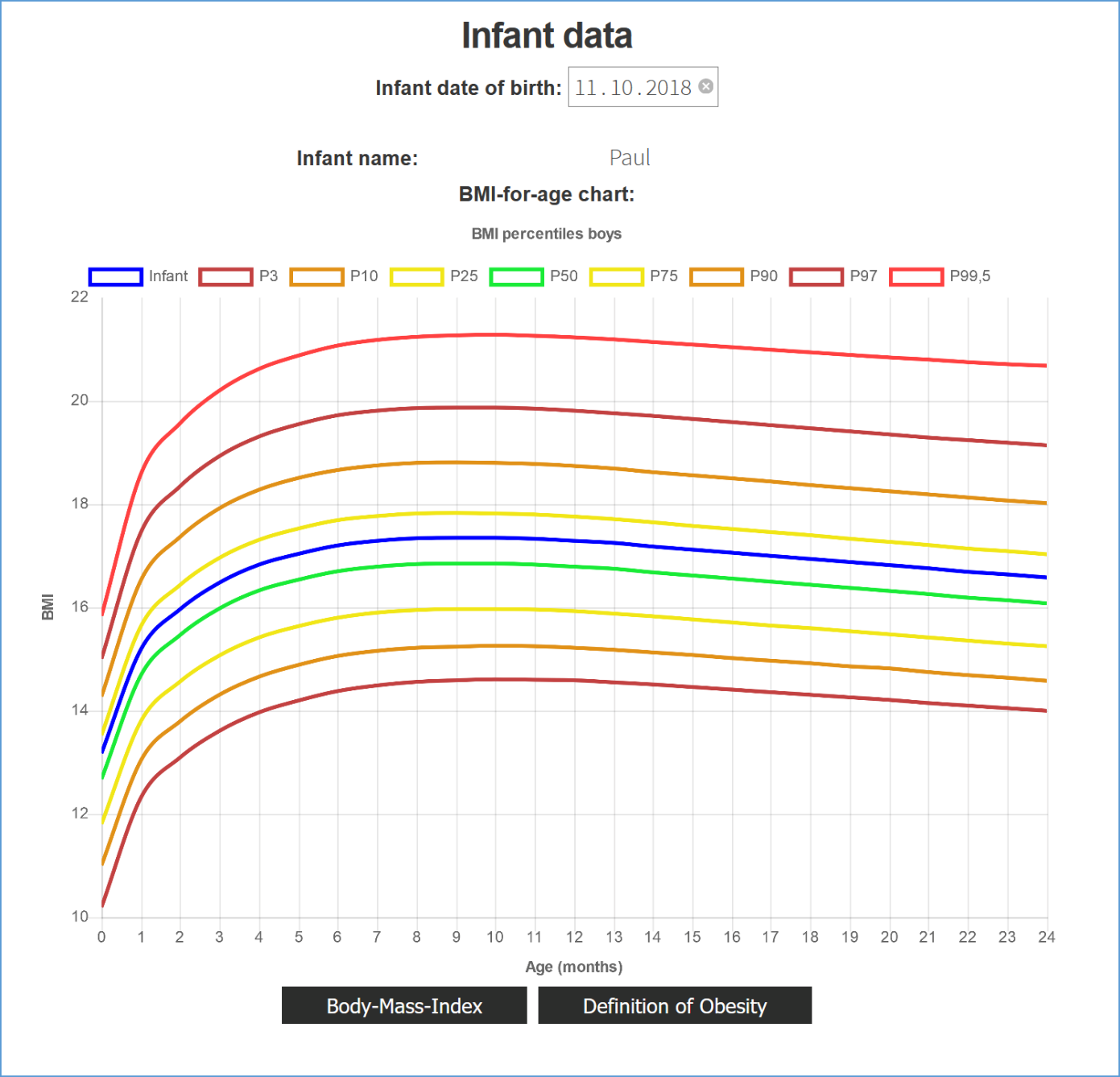


**Figure 2** Infant weight progression using percentile curves displayed in the GeMuKi-Assist counseling tool


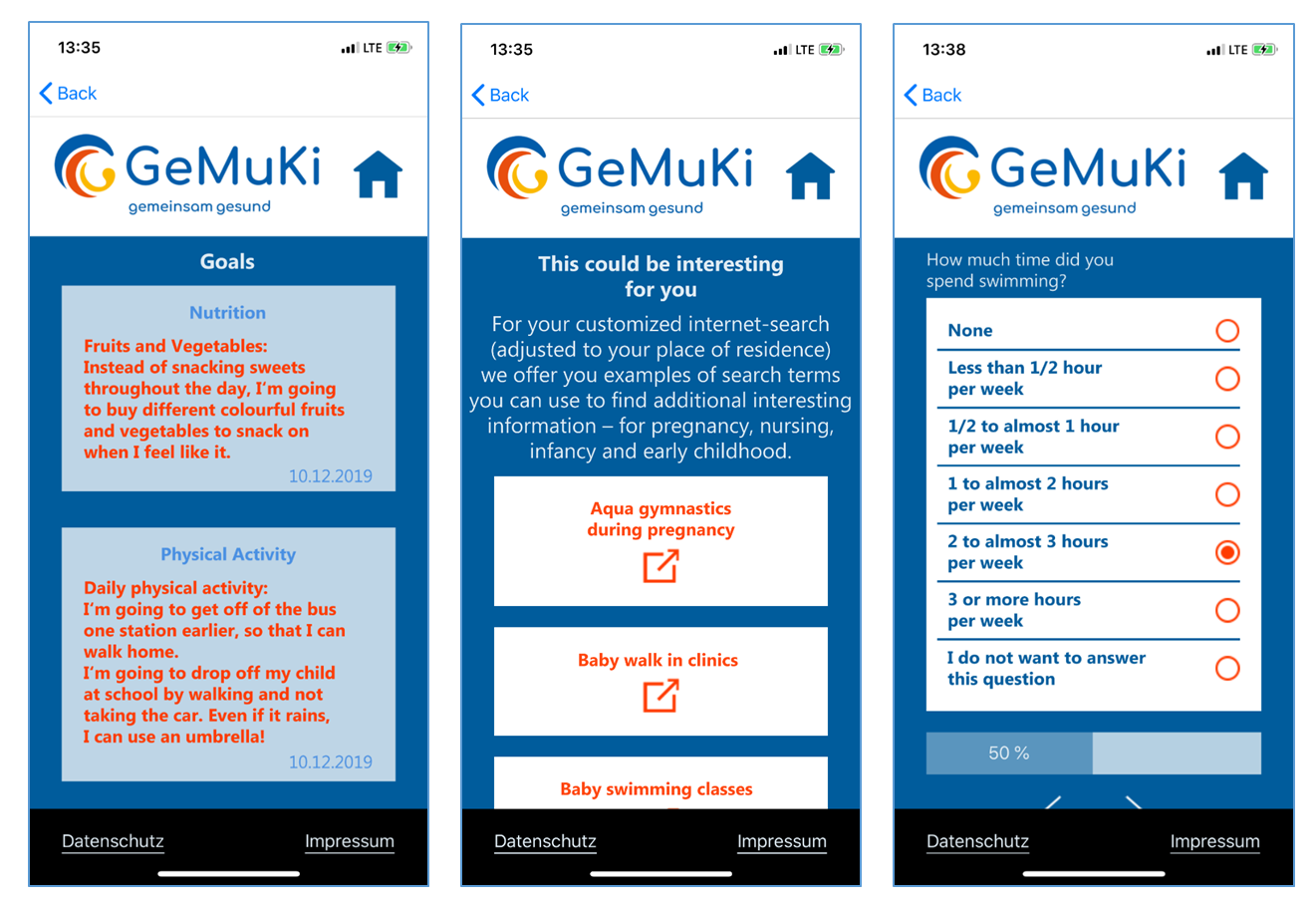


**Figure 3** SMART Goals, automated google keyword searches and survey questions displayed in the GeMuKi- Assist App

**
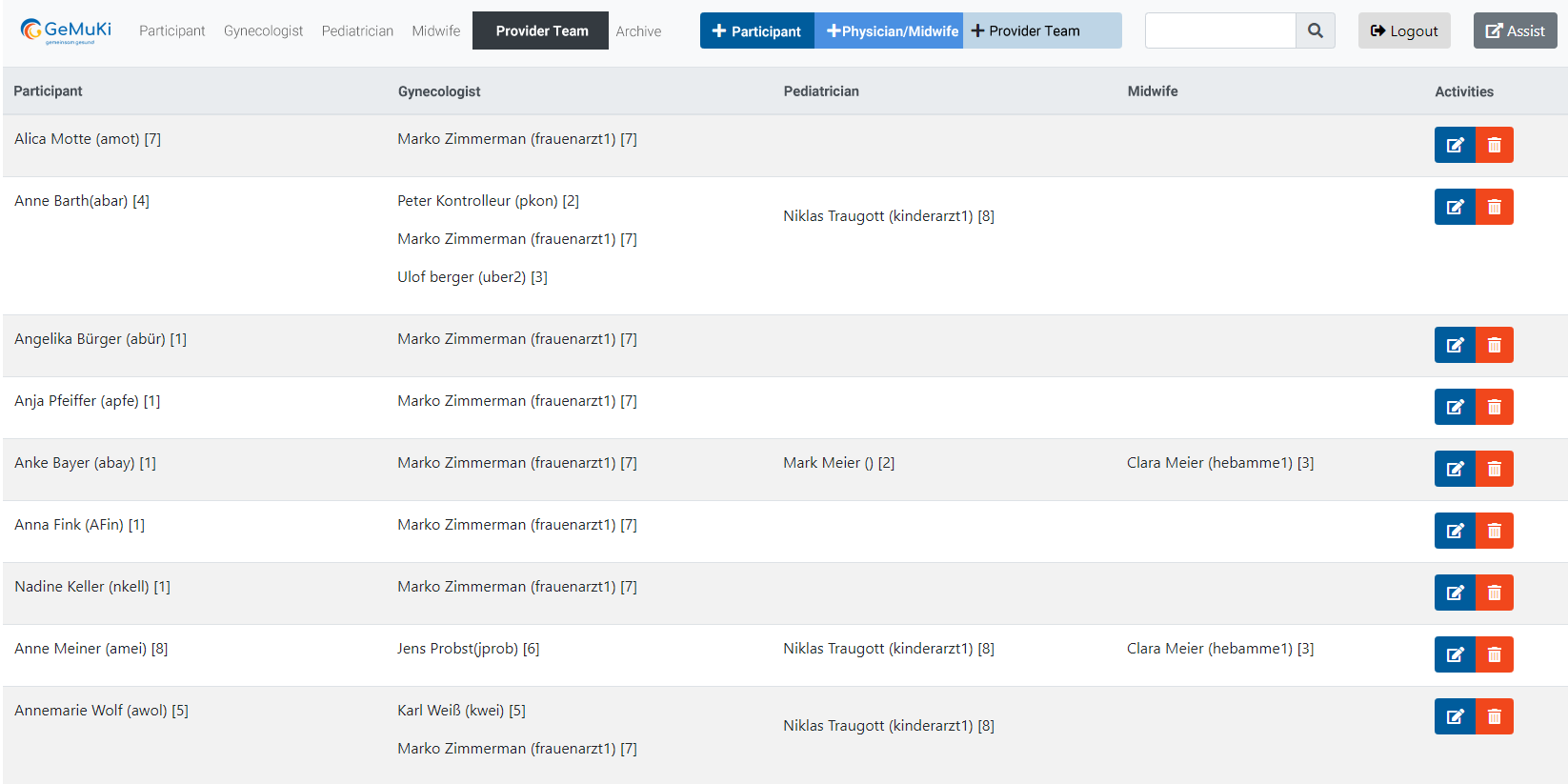
**

**Figure 4**  Overview of study participants and their assigned multi-professional providers displayed in the GeMuKi-Assist Study monitor


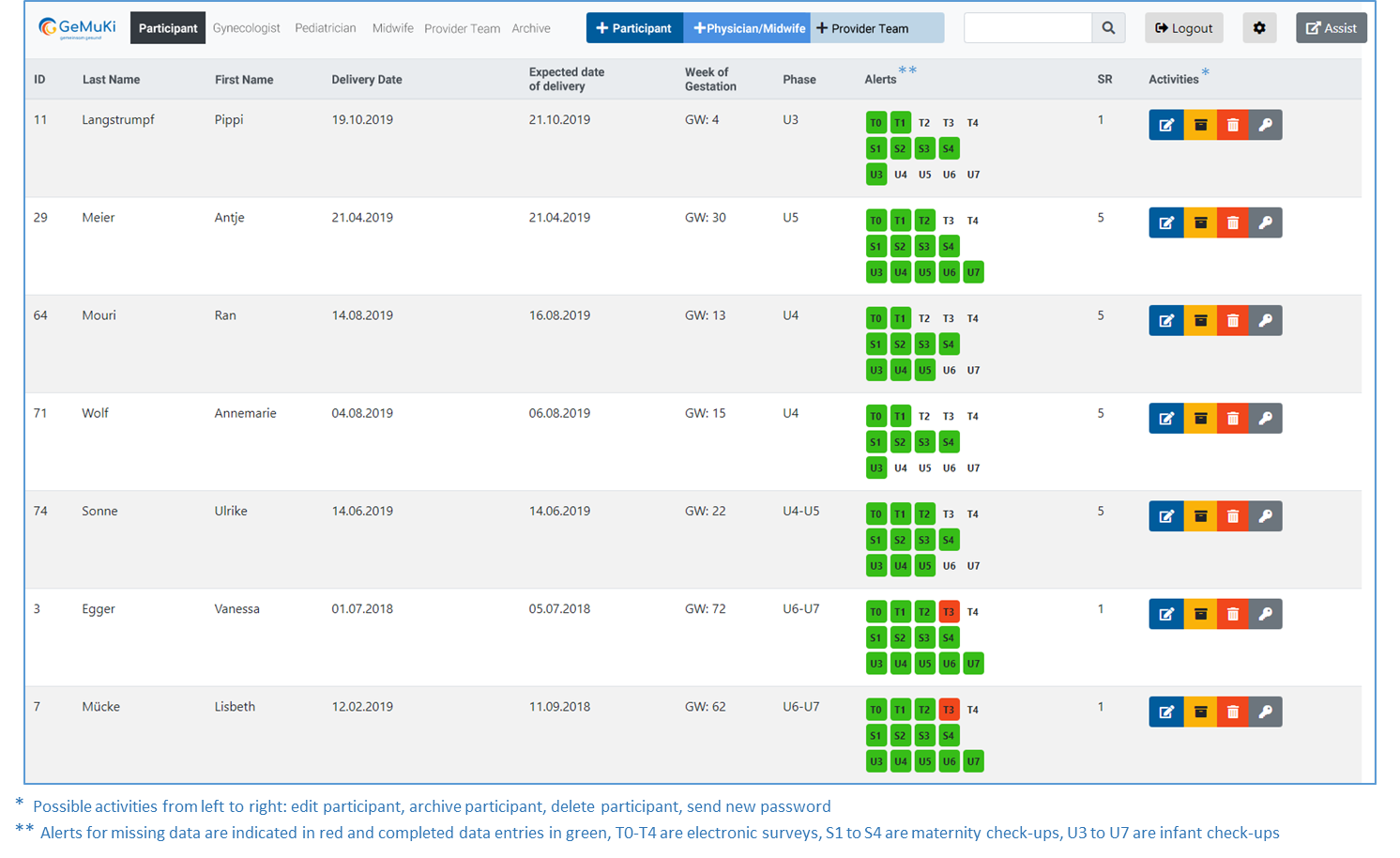
**Figure 5** Overview of data entries by providers and study participant surveys displayed in the GeMuKi-Assist Study Monitor
